# Core–Shell Engineering of One-Dimensional Cadmium Sulfide for Solar Energy Conversion

**DOI:** 10.3390/nano15131000

**Published:** 2025-06-27

**Authors:** Rama Krishna Chava, Misook Kang

**Affiliations:** Department of Chemistry, College of Natural Sciences, Yeungnam University, 280 Daehak-Ro, Gyeongsan 38541, Gyeongbuk, Republic of Korea; drcrkphysics@hotmail.com

**Keywords:** one-dimensional cadmium sulfide, core–shell morphology, heterojunctions, solar-to-fuel conversion

## Abstract

Fabricating efficient photocatalysts that can be used in solar-to-fuel conversion and to enhance the photochemical reaction rate is essential to the current energy crisis and climate changes due to the excessive usage of nonrenewable fossil fuels. To attain high photo-to-chemical conversion efficiency, it is important to fabricate cost-effective and durable catalysts with high activity. One-dimensional cadmium sulfides (1D CdS), with higher surface area, charge carrier separation along the linear direction, and visible light harvesting properties, are promising candidates for converting solar energy to H_2_, reducing CO_2_ to commodity chemicals, and remediating environmental pollutants. The main disadvantage of CdS is photocorrosion due to the leaching of S^2−^ ions during the photochemical reactions, and further charge recombination rate leads to low quantum efficiency. Therefore, the implementation of core–shell heterostructured morphology, i.e., the growth of the shell on the surface of the 1D CdS, which offers unique features such as protection of CdS from photocorrosion, a tunable interface between the core CdS and shell, and photogenerated charge carrier separation via heterojunctions, provides additional active sites and enhanced visible light harvesting. Therefore, the viability of the core–shell synthesis strategy and synergetic effects offer a new way of designing photocatalysts with enhanced stability and improved charge separation in solar energy conversion systems. This review highlights some critical aspects of synthesizing 1D CdS core–shell heterostructures, underlying reaction mechanisms, and their performance in photoredox reactions. Finally, some challenges and considerations in the fabrication of 1D CdS-based core–shell nanostructures that can overcome the current barriers in industrial applications are discussed.

## 1. Introduction

The rising global population and rapid development of industries are the key factors for the energy crisis and environmental pollution. The widespread use of nonrenewable energy sources such as fossil fuels has emerged as a significant challenge to a sustainable environment [1,2,3,4]. Due to its abundance and sustainability, solar energy has been considered the most promising energy source that can rapidly advance the future of renewable energy technologies. In 1972, Fujishima and Honda first reported that the TiO_2_ semiconductor could be used as a photoelectrode to produce a water-splitting reaction [5]. Nevertheless, the corresponding photocatalytic water splitting reaction can only be performed under UV-light absorption (~4% in sunlight) due to the wide bandgap of TiO_2_ (3.2 eV). Therefore, it is essential to develop semiconductor photocatalysts with narrower bandgaps for the realization of excellent activity under visible light. Since then, photocatalysis, a new technology that can directly convert solar energy into chemical energy, has garnered tremendous interest and has been regarded as one of the most promising technologies to solve the issues related to energy and the environment [6,7,8,9]. To develop highly active semiconductor-based photocatalyst materials for water splitting, as well as the photodegradation of pollutants and toxic element wastewater, the optical, electronic, and structural properties of the material have to be carefully investigated. Generally, other features such as morphological architecture, the choice of semiconductor materials, and surface properties should be considered when designing a stable and efficient visible light-responsive photocatalyst material.

**Figure 1:**
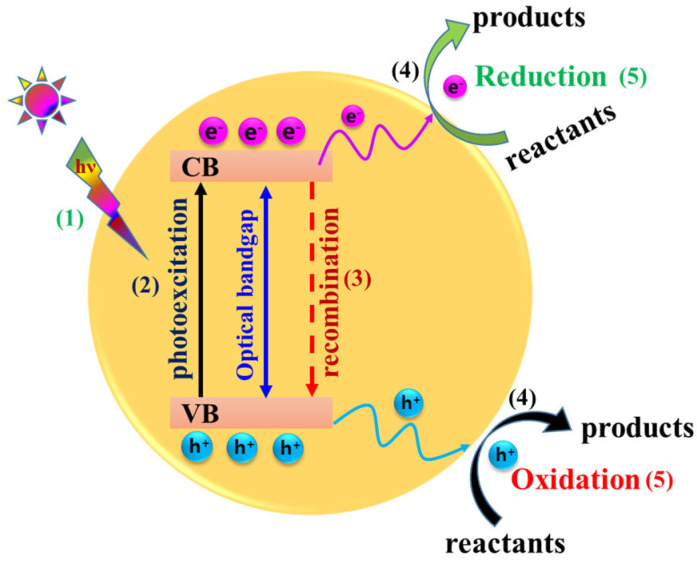
Schematic representation of typical photocatalytic processes on the surface of a semiconductor.

In this regard, semiconductor-based photocatalysis has emerged as an ideal technology in which solar energy as a leading source initiates several photoredox reactions, such as hydrogen (H_2_) evolution, CO_2_ reduction, and pollutant degradation reactions on the surface of a semiconductor. In principle, a photocatalytic reaction on the surface of a semiconductor involves the following steps as mentioned in Figure 1: (1) light absorption by the semiconductor, (2) creation of photoinduced electron–hole pairs, (3) separation and/or recombination of charge carriers, (4) adsorption and desorption of reactants and products, respectively, and lastly, (5) initiation of redox reactions on the surface of the semiconductor.

**Figure 2:**
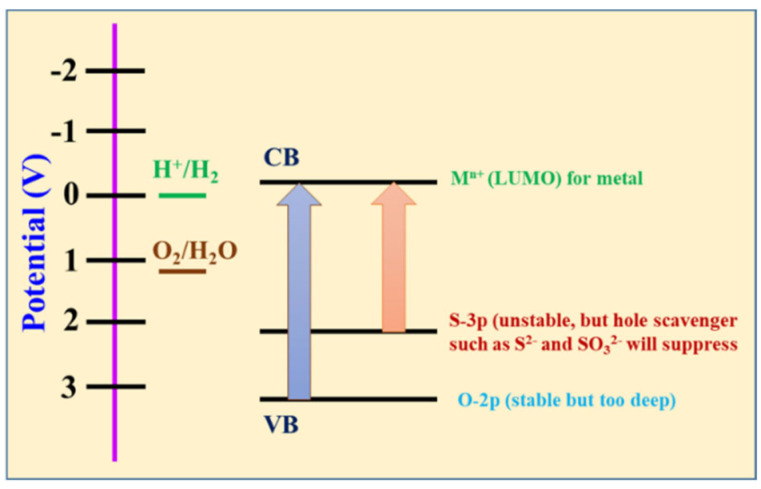
Comparative energy band diagram of metal sulfides and metal oxides, where CB is the conduction band and VB is the valence band.

Among these processes, photoinduced electron–hole pair recombination is the main problem in photocatalytic reactions. During the photoexcitation and photochemical reactions, the electron–hole pairs either migrate to the surface of the semiconductor to participate in the reactions or recombine within the semiconductor, making it ineffective in the form of heat. TiO_2_, while extensively utilized in photochemical reactions due to its incredible properties, faces inherent limitations. The main drawback arises from its relatively large bandgap of 3.2 eV, which can harvest light energy from only a small fraction of the solar spectrum in the ultraviolet region below 400 nm. As a result, TiO_2_-based photocatalysts display poor photocatalytic efficiency under natural sunlight [10]. Based on the pioneering work of Fujishima and Honda on TiO_2_ photocatalysis, substantial efforts have been paid to the other semiconducting materials, such as SrTiO_3_ [11], FeTi_2_O_5_ [12], BiVO_4_ [13], Ag_3_PO_4_ [14], g-C_3_N_4_ [15], CdS [16], ZnIn_2_S_4_ [17], MoS_2_ [18,19], etc., which have been directly applied to exploit solar energy for photochemical reactions. Searching for a suitable semiconductor photocatalyst that can be applied as an effective and stable material is still a challenge for the research community. The intrinsic properties of prepared semiconductor photocatalyst materials can fundamentally direct the overall efficiency of photochemical reactions. Usually, photocatalytic reactions are composed of harsh reaction environments that are highly oxidative or reductive and highly acidic or basic. For this purpose, most of the studied photocatalysts involve metal oxides such as TiO_2_, ZnO, or SnO_2_. However, these metal oxide-based photocatalysts cannot effectively absorb solar light in the visible region due to a higher bandgap (>3.2 eV), confirming their limited practical use for solar energy conversion reactions. Therefore, metal sulfides have drawn attention due to their visible light absorption arising from narrow bandgaps, suitable band positions, and abundant active sites for photochemical reactions [20,21]. Furthermore, the balance band (VB) occupied by the S-3p orbital endows the metal sulfides with a wider photoresponse range and higher carrier concentration [22]. Hence, the combination of metal and sulfide elements results in a metal sulfide with strong electron structure adjustability and surface properties [20]. The bonding of transition metal sulfides is covalent, which can increase the semiconducting properties, but it does have some metallic properties [23]. Most of the sulfide-based photocatalysts are composed of d^10^-configured metal cations since the metal sulfide catalyst conduction bands are composed of d and sp orbitals, while the valence bands involve S-3p orbitals, which are much more negative than O-2p orbitals, as shown in Figure 2.

**Figure 3:**
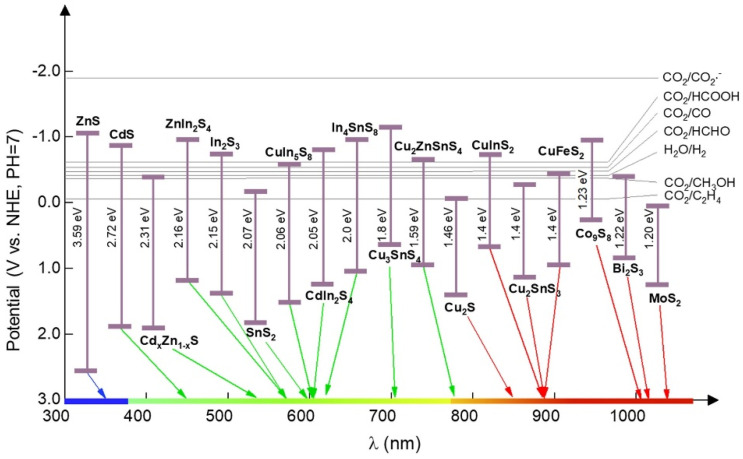
Relative arrangement of band positions of several metal sulfide-based semiconductors. Reproduced with permission from Ref. [30].

Therefore, metal sulfides possess exceptional properties, such as higher photoresponsiveness, higher redox potentials, and a longer lifetime compared to metal oxides. As a result, metal sulfides are attractive candidates for catalysis and as photocatalyst materials in electrocatalytic hydrogen generation, PEC water splitting, environmental remediation, batteries, and sensor fields [24,25,26,27,28]. In addition, several metal sulfides, such as SnS_2_ and MoS_2_, have a layered structure that can help lessen strain generated by volume expansion due to their own high theoretical volume expansion. Moreover, due to high selectivity and sensitivity, the working temperature of metal sulfides is lower than the classical metal oxides; thus, metal sulfide materials play a prominent role in energy and environmental applications [29]. Owing to their excellent properties, metal sulfides are visible light-active photocatalysts based on their bandgap and electronic band structures. The classification is presented in Figure 3.

Among many sulfide-based semiconductor photocatalyst materials, cadmium sulfide (CdS) has received extensive attention and research due to the advantages of a simple preparation method, controlled morphology, strong visible light response, and ability to form stable heterojunctions with other catalysts. Furthermore, the emission studies on CdS also indicate its suitability for applications in optoelectronic and edge emission devices [30]. Several reports validated the photocatalytic efficiency of CdS and CdS-based photocatalyst materials to a greater extent; hence, CdS as a photocatalyst became the standard choice. For example, Wei et al. synthesized rimous CdS nanospheres and then loaded dual catalysts, such as carbon dots and NiS, as co-catalysts for the photocatalytic H_2_ evolution reaction [31]. The unique morphology, internal microstructure, and rough surface of well-dispersed CdS spheres displayed excellent photocatalytic activity. Similarly, we also prepared CdS nanorods via solvothermal reaction, in which cadmium nitrate and thiourea were used as Cd and S precursors, respectively, in the presence of ethylenediamine solvent. The proposed solvothermal route enabled the growth of CdS with a nanorod morphology, and relevant studies were carried out to optimize the size of the nanorod for better photocatalytic H_2_ evolution activity [32]. Next, CdS allomorph junctions with low-energy facet exposure were designed by Zhao et al., where the authors attempted to design CdS with heterojunctions between two crystalline phases: hexagonal and cubic. The resultant hc-CdS successfully achieved a higher photoinduced charge separation rate for the H_2_ evolution reaction. By adjusting the ratio of allomorph junctions within hc-CdS, the photocatalytic H_2_ evolution was boosted. The proposed synthetic strategy was beneficial for designing several photocatalysts with low-energy facets that are not only applicable in the field of photocatalysis but also in other fields, such as electronic and optoelectronic devices [33]. These reports have prompted researchers to design studies on how to synthesize several CdS-based nanostructures. Therefore, intensive efforts have been made to design CdS nanostructures with controllable size, shape, and morphology. The process of introducing anisotropy into a photocatalytic material is considered morphology engineering, which can help to design efficient photocatalysts with improved charge separation processes. Therefore, several methods were adopted by researchers to synthesize CdS nanostructures with different morphologies, such as nanospheres [34], nanoparticles [35], nanosheets [36], hierarchical structures [37], nanorods [38], nanowires [39], nanocubes [40], and quantum dots [41]. The synthesis of such CdS nanostructures with good morphology and crystallinity is able to promote charge separation and thereby improve photocatalytic activity. Among these nanostructures, one-dimensional CdS (1D CdS), such as nanorods or nanowires, has drawn wide attention due to their excellent intrinsic optical and structural properties. In connection with this, several researchers prepared 1D CdS for photochemical reactions. For example, Vaquero et al. synthesized 1D CdS [42] by regulating reaction conditions, such as the temperature and water/thiourea ratio. During the solvothermal reaction, high crystallinity and long nanowires were observed at a reaction temperature of 190 °C; meanwhile, nanorods with poor crystallinity and a short length were observed when the temperature was decreased to 120 °C. Furthermore, the authors confirmed that the water/thiourea ratio in the synthesis process played a minimal role compared to the temperature.

Next, Ullah et al. attempted to grow CdS nanorods with different aspect ratios, grain sizes, and bandgaps via a solvothermal reaction [43]. The obtained CdS nanorods were tested for photocatalytic degradation of organic pollutant methylene blue (MB). The structural, morphological, and photocatalytic tests revealed that the aspect ratio and specific surface area of the CdS nanorods strongly affect the photocatalytic degradation of MB in an aqueous solution. Furthermore, our work on 1D CdS also highlighted the importance of aspect ratio, reaction time, and photoexcited electron–hole recombination rate in a visible light-driven H_2_ evolution reaction [32]. Therefore, 1D CdS became an alternate ideal photocatalyst widely used for several photochemical reactions.

In the past decades, there has been significant interest in designing 1D CdS-based photocatalysts, which mainly involves developing synthesis strategies and investigating characterizations and their applications for photoredox reactions. Until now, only a few review articles on 1D CdS material as photocatalysts have been highlighted [44,45,46]. These review articles only presented the fundamental design of 1D CdS-based composite photocatalysts and their applications in photochemical reactions. Particularly, from the viewpoint of materials design, a clean core–shell heterostructured 1D CdS is useful in designing excellent and highly stable photocatalysts for solar energy conversion reactions.

In this review, the importance of core–shell morphology in 1D CdS nanostructures for charge separation in solar energy conversion reactions is systematically summarized. First, the relevant fundamentals of photocatalysts, working mechanisms, and critical parameters are discussed. Next, the significance of 1D CdS over other dimensional CdS nanostructures is highlighted. Furthermore, various strategies to improve charge separation phenomena in 1D CdS-based heterostructures are presented via defect engineering, metal nanoparticle loading, co-catalyst loading, and heterojunctions. Finally, the advantages of 1D CdS-based core–shell nanostructures, together with their synthesis mechanisms and photogenerated charge transfer, migration, and separation details, are explored. The potential challenges and future opportunities concerning 1D CdS-based core–shell heterostructures for solar-to-fuel conversion reactions are also provided.

## 2. The Emergence of a 1D CdS Photocatalyst and Mechanistic Fundamentals

**Figure 4:**
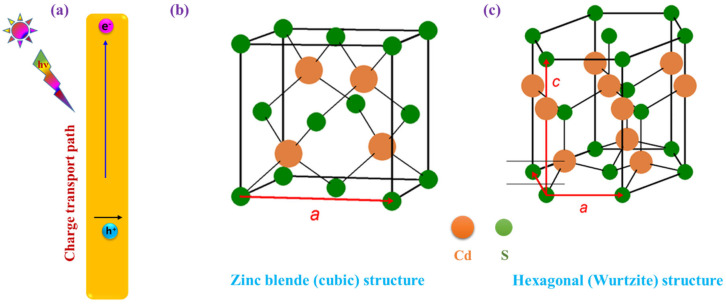
(**a**) Schematic illustration of photoinduced charge separation in 1D CdS. Atomic arrangement of Cd and S atoms in (**b**) zinc blend (cubic) and (**c**) hexagonal (wurtzite) crystal structures.

As an important II-VI semiconductor, 1D CdS with a bandgap of around 2.4 eV has been considered an excellent choice due to its intrinsic optical absorption, electronic band structure, and structural properties. Primarily, 1D CdS nanostructures offer a direct pathway for photoinduced electron transport along the direction of the 1D structure and facilitate separation of the charge carriers (Figure 4a), which is a significant factor in improving photocatalytic activity. Next, due to the higher aspect ratio of the 1D CdS nanostructures, the light absorption and scattering properties are significantly improved in the visible light region. Moreover, when compared to the bulk CdS nanostructures, 1D CdS offers a higher specific surface area and acts as a substrate to load or deposit other components to make heterostructures [47].

According to crystallography, CdS exhibits two types of crystal structures, specifically, hexagonal (wurtzite) and zinc blend (cubic) structures. As shown in Figure 4b, the hexagonal crystal structure is constructed by repeating patterns of ABABAB, while the zinc blend structure is represented by ABCABCABC patterns. Most of the 1D CdS exhibits a wurtzite crystal structure, in which tetrahedrally coordinated S^2−^ and Cd^+2^ planes are arranged alternatively along the c-axis. Moreover, 1D CdS in wurtzite possesses anisotropic growth due to the fact that different crystallographic planes have different surface energies, and their surface polarity and chemical activities may differ [48]. Many researchers have attempted to prepare hexagonal CdS due to its higher catalytic activity compared to cubic CdS. The origin of this activity arises from the high crystallinity of the hexagonal system and due to the distortion of the CdS_4_ tetrahedral unit leading to the creation of an internal electric field that effectively assists in charge separation [49].

As previously mentioned, the 1D nanostructure of CdS is beneficial in improving the visible light harvesting and scattering properties. The linear dimensions of 1D CdS are comparable to several intrinsic physical parameters, such as the wavelength of light (λ), the exciton Bohr radius, the exciton diffusion length, and the mean free path of electrons [50]. The 1D confinement based on the large aspect ratio offers unique properties over the other 2D and 3D nanostructures. The CdS nanostructures with 1D morphology could assist in free passage in the axial direction for charged particles such as electrons/holes and other quantum particles, photons, or phonons [51]. Therefore, with the visible light absorption and long-time charge transport properties endowed, 1D CdS is favorable for the construction of efficient photocatalysts for solar-to-fuel conversion reactions. Thermodynamically, with a direct bandgap of ~2.4 eV, 1D CdS could absorb visible light (400–550 nm), and the positions of VB and CB are much more favorable for oxidation and reduction reactions. These band positions perfectly fulfill the requirements to initiate photochemical reactions such as H_2_ evolution, CO_2_ reduction, N_2_ reduction, and contaminant degradation reactions.

Under visible light irradiation, the VB electrons are excited to the CB, leaving a hole in the VB. Then, the electrons and holes migrate to the CdS surface and reduce or oxidize the adsorbed species, respectively. During this chemical reaction, electron migration is an adequately fast process, while hole transfer is challenging. Therefore, over a period, photoexcited electrons and holes recombine with each other, causing poor activity and making the CdS photocatalyst useless. Next, CdS components are susceptible to photocorrosion during the light irradiation process and show relatively low structural and chemical stability. The photocorrosion is initiated by the excessive gathering of holes on the VB edge, and, as a result, sulfur ions are oxidized to sulfur or sulfate, making CdS an unstable photocatalyst. Therefore, the interior photogenerated holes continuously accelerate the photocorrosion, which leads to the fast deactivation of CdS. The relevant pathways for photocorrosion in CdS are given as follows [52,53,54]:

CdS → h^+^ + e^−^ (1)

CdS + 4h^+^ + 2H_2_O + O_2_ → Cd^2+^ + SO_4_^2−^ + 4H^+^ (2)

O_2_ + 4e^−^ + 2H^+^ → 2OH^−^ (3)

CdS + 2O_2_ → Cd^2+^ + SO_4_^2−^ (4)

The reaction expressed in Equation (4) is the overall photocorrosion in the presence of oxygen. Meanwhile, another type of photocorrosion in the absence of oxygen can be expressed as follows:

CdS + 2h^+^ → Cd^2+^ + S (5)

The above reaction is caused by two holes on the CdS surface, and the possible photooxidized product is sulfur. During the photochemical reactions, the S^2−^ of CdS is readily oxidized, and the toxic Cd^2+^ enters the solution [55]. Therefore, several strategies have been considered to efficiently separate the photoinduced electrons and holes in the CdS material.

## 3. Strategies to Separate Charge Carriers in a 1D CdS Photocatalyst

It is clear that in a 1D CdS semiconductor photocatalyst, the photogenerated electrons in the CB easily recombine with the VB holes, which completely suppresses the photocatalytic activity. This drawback can be overcome by designing proper 1D CdS-based catalysts with improved charge separation efficiency, precisely by doping/defect engineering, metal nanoparticle (NP) loading on a 1D CdS surface, co-catalyst loading, and making heterojunctioned 1D CdS photocatalytic systems.

### 3.1. Doping/Defect Engineering of 1D CdS

The primary and well-established approach to improving the photocatalytic activity of semiconductor-based photocatalysts is the doping process. Generally, doping refers to the introduction of foreign elements into the crystal lattice, which influences electronic and optical properties, thereby enhancing photocatalytic activity. This form of doping, either metals or non-metals, can modify the bandgap of a photocatalyst, causing it to absorb light in extended wavelength regions. Next, doping can regulate VB and CB edge positions to some extent to increase the photoredox and oxidizing powers of the photocatalyst. In addition, doping can also facilitate charge separation and transfer, which are crucial in photochemical reactions. Doping generates extra energy levels within the bandgap of the material that assist in trapping and improving the charge carrier separation, promoting photocatalytic activity. Additionally, doping can offer abundant active sites on the photocatalyst surface to adsorb higher amounts of reactant molecules, thereby increasing the overall efficiency in photocatalytic reactions [20,56]. Therefore, the doping strategy has been adopted by several researchers who have succeeded in increasing the photocatalytic activity of 1D CdS. For example, Zn^2+^-doped CdS nanorods were synthesized by a co-precipitation-hydrothermal strategy. In this work, the obtained Zn_0.5_Cd_0.5_S_1−x_ NRs exhibited a mixed crystal structure of cubic and hexagonal systems. The incorporation of homojunctions and P-induced S vacancies can serve as an effective electron trap to separate the charge carriers and then enhance the pure water splitting reaction efficiency [57]. Beyond Zn^2+^, divalent nickel (Ni^2+^) ions were also doped into the 1D CdS. According to the report by Chen et al. [58], 1D Ni_x_Cd_1−x_S nanorods were obtained by the ethanediamine-assisted solvothermal reaction. In this work, the authors highlighted that the higher amounts of Ni^2+^ doping decreased the aspect ratio of 1D CdS, and the optimal Ni^2+^-doped 1D CdS displayed higher photocatalytic activity toward Rhodamine dye (RhB) degradation. The improved photocatalytic activity is ascribed to the Ni^2+^ doping, which narrowed the bandgap and offered abundant active sites. Furthermore, our recent work also emphasizes that the O-doping and S-defects in 1D CdS increase the photocatalytic H_2_ evolution activity [59]. From these studies, one can emphasize that the doped metal or non-metal ions could assist in tuning the band structures, improve light harvesting from the UV to visible region, restrict the charge recombination rate, and thereby promote photocatalytic activity.

### 3.2. Metal Nanoparticle (NP) Loading onto 1D CdS

**Figure 5:**
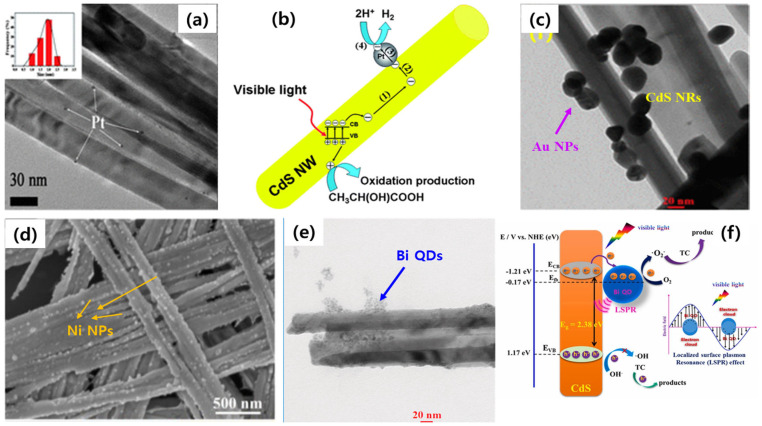
Loading metal NPs onto the surface of 1D CdS: (**a**) Pt NPs, (**b**) charge separation mechanism in 1D CdS/Pt, reproduced with permission from Ref. [61]; (**c**) Au NPs, reproduced with permission from Ref. [62]; (**d**) Ni NPs, reproduced with permission from Ref. [63]. (**e**,**f**) Loading of Bi quantum dots and their charge separation process for a H_2_ evolution reaction and tetracycline degradation reaction, reproduced with permission from Ref. [64].

To improve the charge separation and accelerate the photoredox reactions, the loading of metal nanoclusters or nanoparticles on the surface of 1D CdS is also demonstrated as an efficient approach to increase photocatalytic activity. The loading of noble metal NPs such as Pt, Au, Ag, and Pd has been widely used in improving the performance of semiconductor-based photocatalysts. The loading of metal NPs on 1D CdS, resulting in metal/semiconductor heterojunctions, can offer three benefits. At first, a Schottky junction can be created between the metal NP and CdS. The built-in electric field formed during the equilibrium of the Fermi levels between metal and CdS increases the charge separation. Next, metallic nanoparticles extract the electrons from CdS and act as a co-catalyst in reducing the overpotential for surface reactions. Lastly, metallic NPs could also increase the light-harvesting properties of the resultant photocatalytic system via the surface plasmon resonance effect [60]. For example, the effect of Pt NPs on the surface of 1D CdS was evaluated for an H_2_ evolution reaction [61]. The loading of Pt NPs on the surface of CdS was achieved by several approaches, and among these, a NaBH_4_ reduction in Pt NPs on CdS showed the highest catalytic activity. The well-dispersed and small-sized Pt NPs (10–20 nm) favored the charge transfer efficiently, resulting in the highest activity (Figure 5a,b). Gold NPs (Au NPs) have also been shown to play a significant role in the H_2_ evolution reaction rate of 1D CdS (Figure 5c). The loaded Au NPs not only improved the light-harvesting regions beyond 550 nm but also improved the charge separation via Schottky junctions [62]. Due to the scarcity and expensive nature of noble metal NPs, researchers have also focused on loading inexpensive transition metal-based NPs (nickel and bismuth) onto the surface of 1D CdS [63,64]. The resultant metal/1D CdS photocatalytic systems exhibited higher photocatalytic activity compared to the bare 1D CdS system via the migration of charge carriers from the 1D CdS semiconductor to the metal NPs and subsequently participated in the photochemical reactions.

### 3.3. 1D CdS-Based Heterojunctioned Photocatalytic Systems

**Figure 6:**
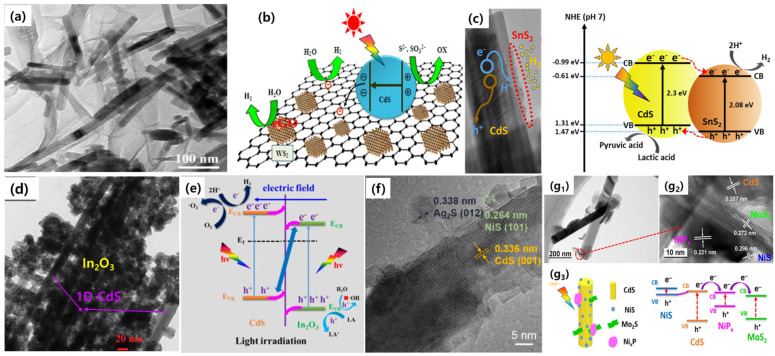
(**a**) TEM image and (**b**) detailed charge transfer mechanism of CdS/WS_2_/graphene composites, reproduced with permission from Ref. [68]. (**c**) TEM image of a CdS/SnS_2_ composite and charge separation process under photoexcitation, reproduced with permission from Ref. [69]. (**d**) TEM image and (**e**) S-scheme-type charge separation in CdS-In_2_O_3_ heterostructures, reproduced with permission from Ref. [70]. (**f**) TEM image of a CdS/Ag_2_S/NiS ternary composite photocatalyst, reproduced with permission from Ref. [71]. (**g_1_**–**g_3_**) TEM images and charge transfer processes in a CdS/NiS/MoS_2_/NiPx quaternary composite photocatalyst, reproduced with permission from Ref. [72].

In addition to the doping and loading of metal NPs onto the surface of 1D CdS, the construction of heterojunctions is also regarded as one of the most promising approaches to improving the charge separation and transfer processes in solar-to-fuel conversion reactions. The preparation of a heterojunctioned photocatalyst system involves the combination of two or more different semiconductors with suitable band structures. After irradiating the heterojunction photocatalysts, charge carriers are spatially separated between the two semiconductors, and as a result, a greater amount of charge carriers are available for photochemical reactions. The main advantages of designing heterostructured photocatalytic systems can be listed as follows: (1) Different semiconducting materials have different band structures and charge transport properties, which can synergistically enhance photocatalytic activity. (2) Construction of heterojunctioned photocatalyst systems absorbs the light radiation in the wider spectral range. (3) The heterojunction photocatalytic system offers chemical stability, especially for sulfide-based materials in long-run photochemical reactions, and avoids the photocorrosion and deactivation of the catalyst [65]. Several researchers focused on designing various semiconductor-based heterostructured systems for photocatalytic applications, which are also highlighted in several review articles [1,7,46,66,67]. Based on these, the utilization of 1D CdS-based heterostructured photocatalyst systems in photocatalytic reactions, such as H_2_ production, CO_2_ reduction, and organic pollutant degradation reactions, has garnered significant attention. For example, Xiang et al. designed a 1D CdS-based ternary photocatalyst in which WS_2_ and graphene sheets are loaded on the surface of CdS nanorods [68]. The resulting CdS/WS_2_/graphene composite system (Figure 6a), with an optimal dosage of 4.2 wt% WS_2_/graphene, displayed an enhanced photocatalytic H_2_ evolution rate of 1842 μmol/g/h. The improved H_2_ evolution rate over bare CdS nanorods could be attributed to the effectively suppressed electron–hole recombination rate, increased interfacial charge separation, and abundant redox active sites (Figure 6b). Tin sulfide (SnS_2_), with a bandgap of around ~2.2 eV, has shown the ability to form heterojunctions with 1D CdS, allowing for potential applications in several research fields. Therefore, SnS_2_ could become an excellent alternative to decorate 1D CdS to improve the optical absorption and charge separation properties. Recent work by Rangappa et al. developed a low-cost and noble metal-free photocatalyst based on 1D CdS-2D SnS_2_ for a water-splitting reaction to generate H_2_ [69]. In this work, the authors decorated exfoliated, layered SnS_2_ nanosheets on the surface of 1D CdS using the physical mixing approach. The obtained CdS-SnS_2_ nanohybrids (Figure 6c), with strong interactions making heterointerfaces, achieved a higher visible light-driven photocatalytic H_2_ evolution rate of 20.0 mmol/g/h with a long-term stability of 20 h. The experimental results indicate that the loaded SnS_2_ nanosheets successfully formed a Type-II heterojunction, and the charge recombination rate was greatly suppressed. Metal oxides such as ZnO, SnO_2_, TiO_2_, and In_2_O_3_ also play a major role in improving the stability and charge separation properties of 1D CdS. For example, our latest work on CdS-In_2_O_3_ heterostructured composites emphasized that In_2_O_3_ grown on the surface of 1D CdS acted as a shield against photocorrosion and thereby promoted photoexcited charge carrier separation [70]. Here, a facile two-step solvothermal and hydrothermal process was adopted to obtain CdS-In_2_O_3_ heterostructures (Figure 6d). The resultant hybrids exhibit a novel S-scheme charge separation path (Figure 6e), where an internal electric field facilitates the separation of charge carriers, thereby preventing the recombination of electrons and holes. This enhanced charge separation then endows the CdS-In_2_O_3_ heterostructures with greater redox powers for H_2_ evolution and tetracycline degradation reactions. The fabrication of 1D CdS-based multicomponent photocatalytic systems also attracted attention as good photocatalysts in several photochemical reactions. In these systems, two or more components, such as metals, sulfides, or oxides, are loaded onto a 1D CdS surface. As a result, multiple charge transfer processes with a higher charge separation rate could be observed. For example, He et al. introduced dual co-catalysts of Ag_2_S and NiS onto 1D CdS via hydrothermal and photodeposition methods [71]. The obtained ternary composite of CdS/Ag_2_S/NiS (Figure 6f) displayed an exceptional H_2_ production rate of 48.3 mmol/g/h. The p–n junction between CdS and NiS and the Schottky junction between CdS and Ag_2_S expedite the transportation of photogenerated holes and electrons, respectively, consequently promoting photocatalytic activity. A step-by-step solvothermal synthesis was reported to obtain a multicomponent system that contains CdS, NiS, MoS_2_, and NiP_x_ [72]. All three components on 1D CdS can be distinguished clearly from TEM and HR-TEM images (Figure 6g_1_,g_2_). The synergetic effects of heterostructures between CdS and NiS and further co-catalyst loading of MoS_2_ and NiP_x_ significantly facilitated the charge separation and improved their lifetimes. As a result, the fabrication of multicomponent photocatalytic systems offers multiple charge transfer paths (Figure 6g_3_), which directly increase photocatalytic activity. In addition to these systems, several researchers have focused on the design of 1D CdS-based heterojunctions for solar-to-fuel conversion reactions. In most of the cases, the heterojunctions between CdS and other components favored the photogenerated charge transfer effectively and improved the lifetime of the charge carriers for participation in photoreactions. Furthermore, the resultant heterojunctions also offer a higher specific surface area to provide abundant active sites. Hence, CdS-based heterojunctions are considered excellent materials for a photocatalytic hydrogen evolution reaction.

Even though extensive research progress has been devoted to the development of 1D CdS-based heterostructured photocatalysts, many issues still need to be considered for real-world applications. First, the fabrication of 1D CdS-based heterostructures involves several synthesis steps that are time-consuming; hence, it is highly essential to design novel and facile methods. Significant challenges continue in the synthesis of such 1D CdS-based heterostructures due to the lack of well-exposed sites and optimized charge separation processes. Second, the irregular contact between CdS and other components limits the applications of 1D CdS-based heterojunctions in solar energy conversion reactions. The uncovered CdS surface in the composite system may degrade in long-run reactions. Third, the fundamental understanding of charge generation, transfer, and migration in the multicomponent photocatalytic system is critical for realizing the real redox and oxidizing sites. Next, the current characterization data determines only eventual efficiency in the photochemical reactions without a detailed understanding of charge transfer in multicomponent systems, which is critical. Therefore, developing a stable 1D CdS-based photocatalytic system with high efficiency and low cost will be the central task for achieving a viable photocatalyst for solar fuel production.

## 4. Core–Shell Heterostructured 1D CdS Photocatalysts

The rational design and integration of multiple functionalities into a single system can create efficient channels for photogenerated charge carrier separation in photochemical reactions, thereby increasing photocatalytic activity. Therefore, the integration of a heterostructured photocatalyst with a proper architecture can open up new prospects that are not possible in a single- or multicomponent system and address critical and fundamental challenges in the design and application of photocatalysts. In this context, core–shell heterostructured photocatalytic systems have been explored and show higher activity and stability compared to their counterparts. A core–shell structure provides a way to maximize the interfaces, which are nearly uniform and well-defined. In contrast to the conventional heterojunctioned composites, in which one component is merely supported on the other, the core–shell architecture increases the contact area at the interfaces. Thus, the development of novel and innovative core–shell photocatalysts is of practical significance in light of their unique properties and applicability to solar-to-fuel conversion reactions. The core–shell nanostructures exhibit ultra-high stability and facile charge carrier separation between the core and shell. The high stability of the core–shell structured system is due to the fact that one component is fully covered by another; the intimate contact between the core and shell facilitates the charge transfer. Therefore, core–shell structured systems became an alternative in several fields, such as solar cells [73], sensors [74], catalysis [75], biomedical applications [76], and photocatalysis [77]. Integrating 1D CdS with unique core–shell morphology provides benefits in solar energy conversion reactions.

### 4.1. Metal Oxides Coated 1D CdS Core–Shell Heterostructures

**Figure 7:**
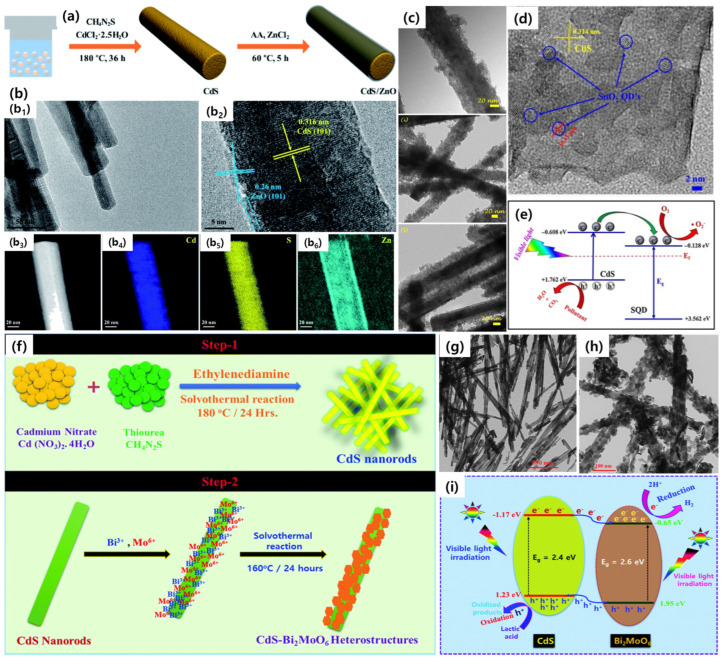
(**a**) Synthesis protocol and (**b**) morphological analysis of CdS@ZnO core–shell nanostructures from (**b_1_**,**b_2_**) TEM images and (**b_3_**–**b_6_**) STEM-EDS elemental mapping profiles, reproduced with permission from Ref. [79]. (**c**,**d**) TEM and HR-TEM images and (**e**) charge separation scheme in CdS@SnO_2_ core–shell structures, reproduced with permission from Ref. [80]. (**f**) Scheme for synthesis procedure, (**g**,**h**) TEM images, and (**i**) Type-II charge transfer in CdS/Bi_2_MoO_6_ heterostructures, reproduced with permission from Ref. [81].

Among the metal oxide semiconductors, TiO_2_, with a wide bandgap of 3.2 eV, is considered the primary photocatalyst and is extensively studied in several photochemical reactions due to its low cost, non-toxic material, and excellent photochemical stability. However, a single TiO_2_ photocatalyst absorbs only UV radiation below 380 nm, and serious electron–hole recombination rates limit the practical applications. Therefore, TiO_2_ is readily capable of making heterojunctions with other semiconductors and enhancing the photocatalytic activity by separating the photogenerated electrons and holes in the heterojunctions. Alternatively, 1D CdS is coated with a highly stable TiO_2_ component that can prevent photocorrosion and improve photocatalytic activity. In this connection, Dong et al. designed CdS@TiO_2_ core–shell structures in which the TiO_2_ shell was meticulously grown on the surface of the CdS nanorods [78]. The facile synthesis process involves controlling the TiO_2_ shell thickness on CdS nanorods via the sol-gel process. In the first step, CdS nanorods were obtained via solvothermal reaction, in which ethylenediamine was the solvent and cadmium acetylacetonate and L-cysteine were used as the Cd and S precursors, respectively. After a reaction temperature of 180 °C for 24 h, the yielded CdS nanorods were used for TO_2_ coating. A simple self-assembled TiO_2_ growth was achieved on the surface of CdS by adding an ethanol solution of titanium (IV) n-butoxide. The thickness of the TiO_2_ shell was controlled based on the amount of Ti precursor. Experimental data reveals that a thickness of 3.5 nm improves the specific surface area, visible light harvesting, and charge separation properties. As a result, higher photocatalytic phenol degradation was achieved. The proposed method allows the tuning of TiO_2_ shell thickness from 3.5 to 40 nm by simply adjusting the Ti precursor. As one of the wide bandgap semiconductors, zinc oxide (ZnO) also attracted great attention in photocatalytic applications due to its comparable properties with those of TiO_2_. Therefore, Sun et al. introduced a chemical deposition method for the selective growth of ZnO on 1D CdS [79]. With this method, the thickness of the ZnO shell and its distribution on CdS NRs can be precisely controlled (Figure 7a,b). The resulting CdS-ZnO core–shell heterostructures, with close interfaces between CdS and ZnO, displayed a photocatalytic H_2_ evolution of 805.5 micromol/h. The band positions of ZnO are suitable to make a charge transfer between CdS and ZnO, and under the photoexcitation, the heterostructure follows a Z-scheme-type charge transfer. Together with the hydrogen spillover effect of ascorbic acid, the CdS-ZnO core–shell structures show improved charge separation properties toward higher photocatalytic activity in H_2_ evolution reactions. Among the metal oxides, SnO_2_ is also regarded as a potential multifunctional photocatalyst. Due to its high chemical stability and significant optical and electrical properties, SnO_2_ can be adopted to coat the surface of CdS, and this opportunity was realized by Babu et al. [80]. In this work, the authors decorated the prepared SnO_2_ quantum dots (QDs) on the CdS NRs via the ultrasonication approach (Figure 7c). The resultant CdS@SnO_2_ core–shell NRs with varying amounts of colloidal SnO_2_ QDs achieved the optimal shell thickness. The effect of SnO_2_ loading on 1D CdS was studied by several characterization techniques and confirmed the formation of core–shell structures (Figure 7d). The optimal concentration of SnO_2_ favored the charge transfer effectively, in which the CB electrons of CdS directly transfer to the CB of SnO_2_ while the holes move in the opposite direction. As a result, the charge recombination rate was suppressed via a Type-II charge transfer (Figure 7e) path, resulting in photocatalytic rhodamine degradation within a 1 h reaction. Very recently, our group also synthesized metal oxide-loaded CdS core–shell nanostructures. In this work, we developed a two-step solvothermal reaction (Figure 7f) for the preparation of CdS-Bi_2_MoO_6_ core–shell heterostructures (CdS-BMO CSHs) [81]. In the first step, CdS NRs were obtained from an ethylenediamine-assisted solvothermal reaction (Figure 7g), and in the second step, BMO nanosheets were precisely decorated on the surface of CdS NRs (Figure 7h). As shown in Figure 7i, the band edge positions of CdS and BMO are suitable for making heterostructures and ensuring effective charge transfer across their interfaces. The intimate contacts between the CdS and BMO allowed the formation of Type-II charge transfer, in which BMO acts as redox sites for the H_2_ evolution reaction. Meanwhile, the accumulated holes in the VB of CdS are consumed by a sacrificial agent and display an excellent photocatalytic H_2_ evolution activity of 6.83 mmol g^−1^ h^−1^, which is six times that of bare CdS NRs. Thus, metal oxide coating on the 1D CdS surface illustrates that the core–shell heterostructures are capable of efficient charge transfer and stability during photochemical reactions.

Lately, choosing a suitable strategy to decorate a co-catalyst on a semiconductor is an important approach to increasing the photocatalytic activity of a photocatalytic system. The spatial decoration of a co-catalyst on 1D CdS is more advantageous in charge separation; hence, it is highly desirable to fabricate core–shell nanostructures that could be used as an alternative to composite photocatalysts. Lu et al. developed a photocatalytic system by integrating a CoO_x_ component as a hole-capturing agent onto 1D CdS NRs [82]. Due to the close contact between CdS and CoO_x_ and the hole-capturing effect of a coated CoO_x_ shell, the photogenerated electrons and holes are accumulated at the CdS and CoO_x_ components, respectively, and as a result, efficient charge separation is achieved. The corresponding photocatalyst displayed an exceptional photocatalytic H_2_ evolution activity of 3.5 mmol/g/h, which is ~43-fold higher than the bare CdS NRs. Therefore, the metal oxide coating on 1D CdS highlights how the designed core–shell structure not only protects the 1D CdS shell from photocorrosion but also contributes to improved visible light-harvesting, charge transportation, and stability, thus increasing the photocatalytic activities.

### 4.2. Metal Sulfide-Coated 1D CdS Core–Shell Heterostructures

**Figure 8:**
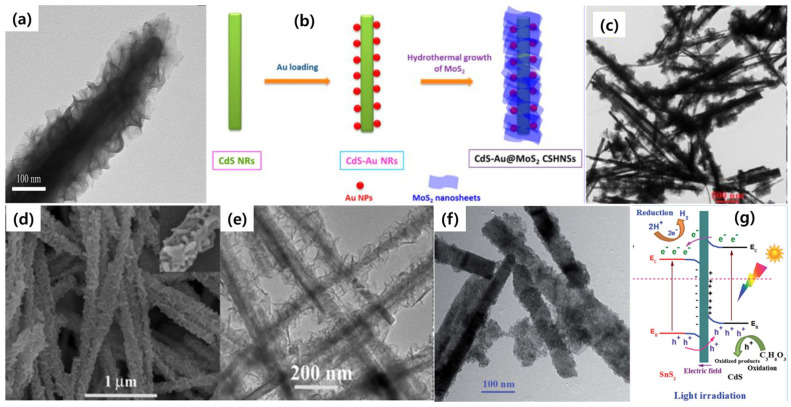
(**a**) TEM image of CdS_-_MoS_2_ core–shell structures, reproduced with permission from Ref. [84]. Synthesis protocol (**b**) and morphology determination by TEM image, scale bar is 500 nm (**c**), reproduced with permission from Ref. [62]. (**d**) FE-SEM and (**e**) TEM images of CdS-CdIn_2_S_4_ heteronanostructures, reproduced with permission from Ref. [85]. (**f**) TEM image and (**g**) understanding the charge separation via S-scheme-type in CdS-SnS_2_ 1D-0D core–shell heterostructures, reproduced with permission from Ref. [87].

Metal sulfides have been widely considered to be photocatalysts in several solar-to-fuel conversion reactions due to their unique properties, such as narrow bandgaps, negative conduction band potentials, easy preparation, and less toxic material. In addition, their tunable crystal structures, optical absorption properties from the visible to infrared regions, and possession of abundant active sites for redox reactions make them an excellent choice as photocatalysts in many photochemical reactions. The fundamental properties and the synthesis protocols for metal sulfides were clearly presented in the latest review article [83]. However, studies on metal sulfide catalysts show that the severe charge recombination and poor electrical conductivity make them a poor choice for photocatalyst applications. Therefore, the fabrication of hybrid heterostructures that involve 1D CdS and metal sulfides could become an excellent choice for practical applications. Among the metal sulfides, MoS_2_, with a layered two-dimensional (2D) structure, offers several advantages, such as fast ionic conductivity and stacked layers, which offer extra sites for the adsorption of H_2_ atoms to facilitate H_2_ evolution reactions. Furthermore, a narrow bandgap of around ~1.8 eV makes it an excellent co-catalyst for visible light-driven photocatalytic applications. When CdS and MoS_2_ are combined into a core–shell heterojunction, the layered sheets of MoS_2_ deliberately coat the surface of CdS NRs, and as a result, improved light harvesting and charge separation can be achieved. For example, Zhao et al. attempted to grow MoS_2_ nanosheets on the surface of CdS nanorods via a solvothermal reaction and successfully obtained CdS-MoS_2_ core–shell nanocomposites [84]. The sulfur source of L-cysteine enabled the formation of a clean MoS_2_ shell under high temperatures (Figure 8a). The careful and optimal loading of MoS_2_ nanosheets enhanced the visible light-harvesting properties of CdS NRs up to infrared regions and thereby generated a higher number of electrons for redox reactions. The resultant photocatalyst showed an enhanced H_2_ evolution activity of 35-fold higher than the CdS NRs, revealing the positive effects of MoS_2_ in H_2_ evolution reactions. Our lab also synthesized CdS-MoS_2_ core–shell heteronanostructures with the assistance of gold nanoparticles. In this work, we successfully grew a MoS_2_ shell via a hydrothermal reaction by using sodium molybdate and L-cysteine as precursors [62]. The careful selection of L-cysteine enabled the growth of a few layers of MoS_2_ on the surface of CdS-Au nanorods, and as a result, a CdS-Au-MoS_2_ core–shell heterostructured (CAM CSHNS) photocatalyst system was obtained (Figure 8b,c). The obtained unique CAM CSHNS comprises a three-dimensional (3D) hierarchical configuration that synergistically improves the rapid electron transfer, and it displays an excellent photocatalytic hydrogen evolution rate. The rational design of core–shell architectures can exhibit the synergic functionality of both core and shell components, which makes them highly efficient solar energy conversion photocatalysts. Wang et al. presented a new solvent-mediated surface reaction growth method for the preparation of CdS nanowire (NW)/CdIn_2_S_4_ nanosheet (NS) 1D/2D architectures [85]. The proposed synthesis route can overcome the traditional physical growth methods; hence, the multidimensional CdS-CdIn_2_S_4_ heteronanostructure with core–shell morphology (Figure 8d,e) has superior charge generation/separation properties and has promising applications in solar energy conversion reactions. Next, tin sulfide (SnS_2_), an n-type semiconductor, has shown promising applications in photocatalysis due to its layered structure and bandgap of around 2.2 eV. Due to the lower toxicity, visible light responsiveness, and earth-abundant nature, SnS_2_ is often considered a co-catalyst in heterojunctioned photocatalysts [86]. Therefore, many efforts have been made to study it; for example, our previous work highlights the growth of SnS_2_ QDs on the surface of CdS NRs as CdS–SnS_2_ 1D-0D core–shell nanostructures for visible light-driven H_2_ evolution reactions [87]. By varying the content of CdS in the reaction, several CdS-SnS_2_ core–shell samples were prepared. According to the characterization data, SnS_2_ QDs with a size of less than 5 nm were evenly distributed and formed a shell layer (Figure 8f) on the surface of CdS NRs. The resultant samples exhibited a higher specific surface area, which offers a greater number of active sites for chemical reactions. After loading SnS_2_ QDs onto CdS, the absorption edge of CdS is redshifted, and their light absorption properties are extended to the infrared regions. Therefore, the CdS-SnS_2_ core–shell structures with improved light-harvesting properties and close interfaces not only improved the charge carrier separation rate but also the photocatalytic H_2_ evolution activity. Usually, SnS_2_ is grown in the form of 2D nanosheets; however, the present synthesis method favors the formation of QDs as a shell, which completely avoids the charge recombination rate and photocorrosion. Based on the band structures and photocatalytic tests, a higher charge separation was achieved via a step-scheme path (Figure 8f) in which the formed internal electric field boosted the charge separation by decreasing the electron–hole pair recombination. Therefore, the reinforced charge transfer between the CdS core and the SnS_2_ shell clearly demonstrates the importance of core–shell structured photocatalysts in solar-to-H_2_ conversion reactions. Moreover, core–shell morphology with controlled thickness and proper shell material selection could be considered for potentially highly efficient photocatalytic systems. Based on these results, the charge transfer mechanism in CdS/SnS_2_ is more efficient when compared to Type-II models, for example, in CdS/Bi_2_MoO_6_ core–shell nanostructures. At first, in the case of Type-II charge transfer, even the photogenerated charge carriers are effectively separated, and the staggered alignment of band structures between CdS and Bi_2_MoO_6_ reduces the overall redox and oxidizing powers of the components, respectively. Meanwhile, an S-scheme model in CdS/SnS_2_ proposes an internal field-assisted charge separation process. Due to the intimate contact between CdS and SnS_2_, the electrons and holes move from one component to the other, and after some time, an electric field is created at the heterojunctions, which further restricts the movement of the charge carriers. As a result, the useless electrons and holes could be eliminated; under the photoirradiation process, the electrons and holes with strong redox and oxidizing powers are retained for subsequent photochemical reactions. Therefore, designing 1D CdS-based S-scheme-type charge transfer models could be of interest in realizing highly efficient photocatalytic systems.

### 4.3. Carbon-Based Material-Coated 1D CdS Core–Shell Heterostructures

**Figure 9:**
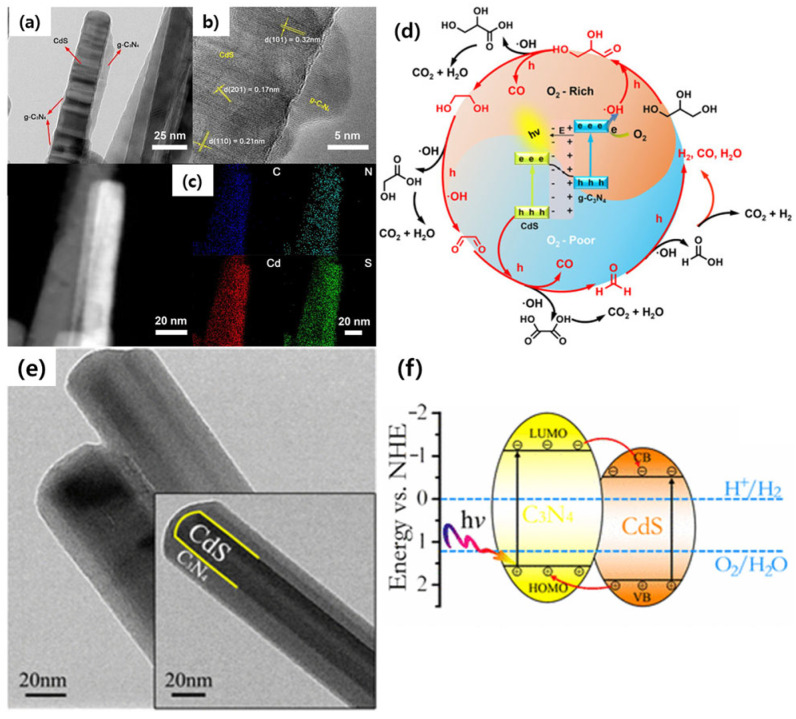
(**a**–**c**) TEM, HR-TEM, and mapping profiles; (**d**) Z-scheme-type charge transfer in CdS-g-C_3_N_4_ core–shell heterostructures, reproduced with permission from Ref. [88]. (**e**) TEM image and (**f**) Type-II charge separation process in CdS/C_3_N_4_ core–shell heterostructures, reproduced with permission from Ref. [89].

Significant progress in the fabrication of core–shell nanostructured photocatalysts has been made in terms of stability and activity. As mentioned in the previous sections, metal oxides and metal sulfides have proven to be good shell materials that successfully shield the core CdS from photocorrosion and improve the stability and activity to a greater extent. Recently, carbon-based materials have also been explored as good photocatalytic materials due to their excellent physicochemical properties, such as high electrical conductivity, high electron mobility, greater specific surface area, and optical absorption properties in wider regions. Particularly, graphitic carbon nitride (g-C_3_N_4_) or graphene oxide (GO/rGO) components act as co-catalysts/charge mediators in the heterostructured photocatalysts. Since g-C_3_N_4_ is a polymeric semiconductor photocatalyst with a bandgap of 2.7 eV, its suitable band structures make it possible to combine with the 1D CdS. Therefore, there is a possibility of charge separation and then higher photocatalytic activity. For example, Zhou et al. designed a core–shell structured photocatalyst that contains 1D CdS as the core and g-C_3_N_4_ as the shell material [88]. In this work, a self-assembly process was designed to obtain a CdS@C_3_N_4_ core–shell photocatalyst. At first, g-C_3_N_4_ nanosheets were obtained via HNO_3_-assisted exfoliation, and then CdS nanorods were wrapped in the g-C_3_N_4_ nanosheets. The resultant CdS@C_3_N_4_ core–shell heterojunctioned photocatalyst (Figure 9a–c) was utilized in the photo-reforming reaction to promote CO evolution. A core–shell structured CdS@g-C_3_N_4_ with a Z-scheme charge separation path (Figure 9d) facilitated the adsorption and activation of O_2_ molecules and accordingly improved the CO evolution activity. Similarly, the photocorrosion problem of 1D CdS was investigated by coating with a g-C_3_N_4_ shell, and as a result, the obtained g-C_3_N_4_/CdS hybrid photocatalyst was considered for a visible light-driven H_2_ evolution reaction [89]. The finely-coated 1D CdS with a g-C_3_N_4_ shell (Figure 9e) exhibited excellent photostability during the H_2_ evolution reaction, and this phenomenon is attributed to the well-matched band structures between CdS and g-C_3_N_4_ with further close contacts enabling charge separation via the Type-II scheme (Figure 9f). The synergetic effect of the core–shell configuration and heterojunctions, CdS/g-C_3_N_4_, could prove to be a promising candidate for photocatalytic applications. Thus, the coating of suitable abundant carbon-based materials on 1D CdS may represent a suitable design for proficient catalysts for photoredox reactions.

Therefore, in recent years, research on photocatalyst designs is no longer limited to the simple procurement of materials because the synthesis strategies of these materials have also gained more attention. Based on several research works, it has been found that different preparation methods yield different nanostructures and can also regulate the morphology of the obtained products. In the preparation process, morphology is directly controlled by reaction parameters, such as time and/or temperature; as a result, novel nanostructures could be designed for several photocatalysis applications. Hence, it is an appealing choice to regulate the morphology, especially in core–shell heterostructures, thereby increasing the photocatalytic activity of the designed photocatalysts. Mostly, hydrothermal or solvothermal methods are the best choice for the preparation of excellent photocatalysts. These methods refer to the process of dissolving and crystallizing the soluble reactants in water or organic solvents, respectively. As noted, temperature, pressure, and reaction time in the hydrothermal/solvothermal process can define the morphology properties of 1D CdS photocatalysts. Consequently, the selection of suitable reaction conditions is of great importance to obtain a favorable morphology and reproducibility, thus optimizing the performance of photocatalysts.

## 5. Conclusions and Future Challenges

In summary, the current review article explores the superiority of 1D CdS core–shell nanostructures over composite photocatalysts. The categories of 1D CdS-based heterostructures, along with their synthesis protocols, charge separation pathways, and photocatalytic performances, were thoroughly discussed and analyzed. Noticeably, 1D CdS has proven to be an excellent visible, active photocatalyst and suitable for designing advanced photocatalysts for several photochemical reactions. Numerous approaches have been utilized to boost the photocatalytic activity of 1D CdS, including defect/dopant engineering, co-catalyst loading, and heterojunctions. Yet some performance-related problems remain, e.g., the most serious problem of photocorrosion making the 1D CdS inactive. Therefore, designing 1D CdS-based core–shell nanostructures solves the problem to a greater extent.

Hence, this review article helps to outline the innovative design of 1D CdS-based core–shell nanostructures for solar energy conversion reactions. Here, the optimized core–shell design model is able to provide several advantages, such as improved light harvesting, optimization of heterointerfaces between core and shell components, and charge separation via efficient paths (Type-II, direct Z-scheme, and S-scheme), which directly results in enhanced photocatalytic activity. Also, from this review, it is clear that the design of S-scheme charge transfer-based 1D CdS photocatalysts could offer more space in designing excellent visible light-driven photocatalysts.

In the future, more attention should be directed toward studying the relationships between structures, functions, and the photocatalytic reaction mechanism of 1D CdS-based core–shell nanostructures. Furthermore, it is concluded that controllable synthesis and fine-tuning of shell thickness play a major role in promoting charge separation and photocatalytic activity. Despite the promising applications of 1D CdS-based core–shell nanostructures in solar-to-fuel conversion reactions, challenges persist, instigating further studies into the following areas:

(i) The main feature in the photochemical reactions is the charge generation and migration phenomenon, which dictates the final activity of the designed photocatalysts. Therefore, understanding the photoinduced charge separation between the core and shell components is essential for optimizing the architecture and then enhancing the activity.
(ii) Detailed investigations into morphology, crystal structure, defects, and reactive sites are crucial for achieving high-performance photocatalysts in several redox reactions. In this regard, precise control is essential in realizing the 1D CdS-based core–shell synergized nanostructures.
(iii) To understand the synergetic interactions of the core and shell, extensive exploration of in situ characterizations, for example, in situ X-ray photoelectron spectroscopy (in situ XPS), Raman spectroscopy, X-ray absorption near edge structure (XANES), etc., is expected.
(iv) Even though the shell layer on 1D CdS offers significant physical protection from photocorrosion, the interactions between the 1D CdS core and shell should be stronger to restrict the leaching of Cd or S ions in the photocatalysts.
(v) Synthesizing the 1D CdS core–shell nanostructures via a cost-effective approach and scaling up strategies also play a major role in utilizing them in sustainable energy technologies.
(vi) In addition, advanced DFT calculation and simulation/modeling studies should also be adequately utilized to simulate and validate the growth formation and internal mechanisms of 1D CdS-based core–shell heterostructures in future research.

Hence, part of future research should be focused on the comprehensive fundamental understanding of relationships between core and shell components and further structural features dependent on photoredox characteristics, which would enable one to accurately tailor the individual components, their interfaces, and overall photocatalytic systems for both solar energy conversion and optoelectronic devices that are made from 1D CdS-based core–shell nanostructures.
